# Genome-wide evolutionary characterization and expression analysis of the *AT-HOOK MOTIF CONTAINING NUCLEAR LOCALIZED* gene family in *Brachypodium distachyon*

**DOI:** 10.1093/g3journal/jkaf275

**Published:** 2025-11-17

**Authors:** Jessica Ortiz-Eriamiatoe, Xin Xin, John A Hadish, Michael M Neff

**Affiliations:** Department of Crop and Soil Sciences, Washington State University, Pullman, WA 99164, United States; Molecular Plant Sciences Program, Washington State University, Pullman, WA 99164, United States; Department of Crop and Soil Sciences, Washington State University, Pullman, WA 99164, United States; Molecular Plant Sciences Program, Washington State University, Pullman, WA 99164, United States; Department of Crop and Soil Sciences, Washington State University, Pullman, WA 99164, United States; Department of Crop and Soil Sciences, Washington State University, Pullman, WA 99164, United States; Molecular Plant Sciences Program, Washington State University, Pullman, WA 99164, United States

**Keywords:** Brachypodium distachyon, AHL family, AT-hook motif, phylogenetics, synteny, expression profile, comparative genomics, plant evolution, DUF296/PPC domain, crop improvement

## Abstract

Plants adapt to diverse environments through complex gene regulatory networks, with the *AT-HOOK MOTIF CONTAINING NUCLEAR LOCALIZED (AHL)* gene family playing a crucial role. This research identified and examined the *AHL* gene family within *Brachypodium distachyon,* a model plant for Pooideae grasses including essential cereal crops. AHL proteins are conserved across land plants, suggesting an ancient origin and fundamental importance in plant development and adaptation. *B. distachyon* is an efficient research model for monocot studies due to its compact genome, short life cycle, and genetic manipulation compatibility. While *Arabidopsis thaliana* has consistently served as a valuable model for studying the *AHL* gene family, understanding their function in monocots, particularly grasses, is essential for crop improvement. The conserved evolutionary history of AHL proteins makes them an excellent target for comparative genomic research across eudicots and monocots systems. Although *AHL* functions have been studied in *A. thaliana*, their roles in Pooideae grasses remain largely unknown. This research identified 22 *BdAHL* genes classified into 2 monophyletic clades and 3 protein types based on conserved domains. By characterizing *BdAHL* gene structure, phylogeny, expression, and potential protein interactions, this study lays the groundwork for future functional analyses of Pooideae grasses. *BdAHL* expression profiles across different tissues and global coexpression patterns were also examined. These findings provide a foundation for future research into specific *AHL* gene functions in *B. distachyon* growth, development, and stress responses, potentially enhancing our understanding of *AHL* function in other Pooideae grasses and aiding crop improvement strategies.

## Introduction

As sessile organisms, plants have developed mechanisms to respond to environmental changes, which help them survive and reproduce. Plant response mechanisms become regulated through gene expression changes triggered by both biotic and abiotic stress stimuli. The overall growth and development of plants is significantly influenced by a range of external factors including light, temperature, and photoperiod (for review see Braidwood et al. 2014). Environmental adaptation requires the essential process of controlling gene transcription. The interaction of transcription factors with complex gene regulatory networks enables plants to adjust their responses to diverse and fluctuating environments.

The DNA-binding *AT-HOOK MOTIF CONTAINING NUCLEAR LOCALIZED* (*AHL*) gene family acts as fundamental regulators of overall plant growth and development (Fujimoto et al. 2004; Zhao et al. 2014). AHL proteins exist across a wide spectrum of terrestrial plant lineages from mosses to flowering plants highlighting their ancient origin within the angiosperm lineage while demonstrating their crucial role in plant growth and adaptation mechanisms (Zhao et al. 2014). Research into gene families can provide valuable information about how major angiosperm groups diversified (for review see Wendel 2000). Studying AHL gene family members in both monocots and eudicots can help scientists identify groupings specific to evolutionary clades and understand gene duplication patterns. The unique developmental paths seen after the monocot–eudicot divergence resulted from differential gene duplication events and functional divergence within the gene families (for review see Guo et al. 2023). The evolutionary development of the AHL gene family as well as its role in flowering plant diversification requires comparative genomics analysis of orthologous and paralogous AHL genes through phylogenetic analyses.

AHL proteins contain 2 conserved domains, the AT-hook and the plant and prokaryote conserved domain (PPC/Domain of Unknown Function 296 (DUF296)). AHL proteins interact with each other and can influence the recruitment of RNA polymerase to DNA by physically binding to regulatory elements thereby modulating gene expression (Matsushita et al. 2007; Ng et al. 2009; Yun et al. 2012; Zhao et al. 2013). The AT-hook motif allows AHL proteins to physically interact with specific DNA sequences within the regulatory regions of target genes. The core sequence of the AT-hook motif, Arginine–Glycine–Arginine (R–G–R), adopts a specific conformation upon binding the minor groove of AT-rich DNA, enabling a tight interaction (Huth et al. 1997). Unlike the AT-hook containing HMGA proteins in mammals, AHL proteins always include 1 or 2 AT-hook motifs as well as a PPC/DUF296 domain.

The AHL protein family is characterized by the presence of a PPC/DUF296 domain, which facilitates both homo- and hetero-oligomerization, notably trimer formation (Fujimoto et al. 2004; Zhao et al. 2014). The PPC/DUF296 domain possesses a conserved 6-amino-acid region, Glycine–Arginine–Phenylalanine–Glutamate–Isoleucine–Leucine (G–R–F–E–I–L), across all *Arabidopsis thaliana* (Arabidopsis) AHL proteins (Zhao et al. 2013). Distinct from many transcriptional regulators, AHL proteins interact with the minor groove of DNA. It has been hypothesized that AHL complex binding to AT-rich regions induces conformational changes in DNA, potentially mediating the juxtaposition of distal regulatory elements to promote the assembly of transcriptional complexes (Huth et al. 1997; Zhao et al. 2013). Further research on the specific target genes regulated by AHL proteins and their downstream effects on plant physiology will provide deeper insights into the intricate mechanisms by which plants orchestrate their responses to a dynamic environment.

Understanding the genetic mechanisms governing physiological processes in grasses (Poaceae) is crucial for optimizing crop yields and developing stress-resistant cultivars, especially considering their critical role as primary food sources and biofuel contributors (for review see Bevan et al. 2010). The study of AHLs in Pooideae grasses represents a valuable opportunity to unlock the mechanisms controlling growth development and stress responses among important crop and temperate grasses. Since AHLs perform established roles in Arabidopsis research work on Pooideae grasses may uncover new information beneficial for crop enhancement and stress resistance. Although, several observations have been published pertaining to the use of *AHL* genes for crop development (for review see Martinčová and Soukup 2025), research has yet to determine the functions of AHLs in *Brachypodium distachyon* (Brachypodium) as well as in any other species of the Pooideae subfamily. Brachypodium has emerged as a valuable model organism due to its close phylogenetic relationship to economically important grass species such as rice, wheat, sorghum, and temperate grasses (for review see Draper et al. 2001). This close kinship allows researchers to leverage Brachypodium for studying gene function and regulation in Pooideae species, ultimately facilitating the discovery of beneficial genes, particularly from stress-tolerant plants.

For more than half a century, Arabidopsis stands out as an excellent model organism because of its beneficial qualities together with extensive resources and proven research methodologies to investigate gene function evolution. The system serves as a robust tool to explore gene evolution by analyzing gene modifications throughout time which affect adaptation and diversity in plant evolution. Like Arabidopsis, Brachypodium fulfills the need for a small, diploid organism with several advantages: ease and affordability of cultivation under controlled conditions, compatibility with a comprehensive suite of modern molecular tools, and rapid life cycle (Draper et al. 2001). By combining the strengths of both models, scientists can gain a deeper understanding of how genes evolve and how these changes shape plant life. Arabidopsis and Brachypodium are complementary model systems for functional genomics in plants.

Here, we investigate the BdAHL gene family in Brachypodium to expand our understanding of AHL gene function throughout different plant species while addressing current knowledge gaps. Our analysis of BdAHL family members includes assessments of gene structure, phylogenetic relationships, chromosomal location, gene collinearity, gene duplication events, conserved motifs, and protein–protein interactions. We analyze the BdAHL gene expression profiles in different Brachypodium tissues while also assessing gene coexpression patterns on a global level. These findings provide a foundation for continuing research to uncover the specific functions of AHL genes in Brachypodium growth, development, and stress responses while examining other Pooideae grasses.

## Methods

### Genome-wide identification and phylogenetic relationship of analyses of *BdAHLs*

Identification of the *AHL* gene family in Brachypodium was conducted using 29 Arabidopsis AHL protein sequences (The Arabidopsis Information Resource; Bernardini et al. 2015) as queries for BLASTp and tBLASTn searches against protein and genome databases using Phytozome v13 (E-value < 1e^−5^) (Goodstein et al. 2012). Candidate proteins from *Oryza sativa*, *Zea mays*, and *Sorghum bicolor*, also identified by using Arabidopsis as query protein sequences, were collected from NCBI (NCBI Resource Coordinators 2016). Redundant sequences were manually removed. Further confirmation was performed using InterProScan (Jones et al. 2014) to ensure the presence of both AT-hook motif(s) and PPC/DUF296 domain within the identified sequences. Only intact genes containing both domains were used for subsequent in-depth searches within Phytozome v13. The physico-chemical parameters of proteins including isoelectric point, net charge, and average molecular mass were calculated using Prot pi Protein Tool online software (https://www.protpi.ch/Calculator/ProteinTool) (Josuran et al. 2025). Subcellular localization prediction analysis was performed using LOCALIZER 1.0.4 online software (https://localizer.csiro.au/) (Sperschneider et al. 2017).

### Phylogenetic analysis of AHL family members and orthologous groups identification

All sequence alignments and phylogenetic analyses were performed using Molecular Evolutionary Genetics Analysis software version 11 (Tamura et al. 2021). The Jones–Taylor–Thornton (JTT) matrix-based model was used with the maximum likelihood method to determine evolutionary history. The evolutionary relationships of the analyzed taxa were depicted through a bootstrap consensus tree that was created with 1,000 replicates. Branches with partitions that appeared in fewer than 50% of the bootstrap replicates received collapsed representation. The percentage of replicate trees where the associated taxa clustered together in the 1,000 replicate tests is shown next to the relevant branches as bootstrap support values. Gene structure and motif identification data visualization was performed with TBtools (Chen et al. 2020) and MEME Suite 5.5.7 (https://meme-suite.org/meme/) (Bailey et al. 2015). Sequence logo analysis was conducted using the MEME Suite 5.5.7. Protein sequences were used for identification of orthologous AHLs across different plant species. Orthologous Brachypodium AHLs were further analyzed and confirmed using PANTHER 19.0 (https://www.pantherdb.org/) (Thomas et al. 2003) (Supplementary Table 1).

### Synteny and collinearity analyses

To visualize the genome conservation of *A. thaliana* and *B. distachyon*, we conducted a synteny analysis using the Circoletto tool (bat.infspire.org/circoletto/) (Darzentas 2010). Gene family expansion in plants is driven by tandem and segmental duplications. Segmental duplications, resulting from polyploidy, and tandem duplications, caused by short-fragment crossovers (Cui et al. 2006; Liu et al. 2014), contribute to genome evolution. To analyze *BdAHL* gene duplications, we employed BLASTp searches and the MCScanX function within TBtools (default parameters). The MCScanX Diamond output was used to quantify *B. distachyon* genome replication events. Duplication types for each *BdAHL* gene were determined using the MCScanX. The Ka, Ks, and Ka/Ks ratios of tandem repeat *BdAHL* gene pairs were calculated using the Ka/Ks calculator in TBtools (Chen et al. 2020). Synteny of *BdAHL* genes with *AHL* genes from *A. thaliana* was visualized using the One-Step MCScanX function of TBtools. Dual collinearity plots were generated to illustrate synteny.

### Dating gene duplication events in Brachypodium

The Plant Genome Duplication Database (PGDD) (Lee et al. 2012) was used to search for segmental duplications that contain *AHL* genes. The analysis assessed Ks values, which serve as a measure of synonymous substitution rates at synonymous sites (Ks) in duplicated gene pairs. Ka values, which serve as a measure of nonsynonymous substitution rates at nonsynonymous sites (Ka) in duplicated gene pairs, were also calculated. Within PGDD, genes were considered segmentally duplicated if they met the following criteria: Segmentally duplicated genes had Ks values between 0 and 1 and at least 3 shared homologous regions in the same plant species. The rate of synonymous substitutions specific to cereals including *B. distachyon* (Blanc and Wolfe 2004) allowed us to estimate a segmental duplication rate of *AHL* genes at 6.5 × 10^−9^ genes per year. The approximate age (*T*) of these gene duplication events was calculated by applying the formula *T* = Ks/2λ described by Song et al. (2014). TBtools was used to calculate this data (Chen et al. 2020).

### Protein–protein interaction analysis of *Bd*AHLs and *At*AHLs

Predicted protein–protein interaction networks were constructed using STRING v.12 (Szklarczyk et al. 2023). Confirmed *Bd*AHL (22 nodes) and *At*AHLs (29 nodes) protein sequences were used to ensure only query proteins were included. Networks were generated selecting all active interaction sources and setting a minimum interaction score of 0.400. Network k-means clustering was applied using 2 clusters.

### Gene expression matrix construction

Publicly available *B. distachyon* RNA-seq data was downloaded and processed using Gene expression matrix (GEM)maker (Hadish et al. 2022). Datasets were identified on the NCBI SRA database (NCBI Resource Coordinators 2016). GEMmaker was run using the Kallisto (Bray et al. 2016) pipeline with the *B. distachyon* genome version 3.0 and annotation version 3.2 (International Brachypodium Initiative 2010) downloaded from Joint Genome Institute (JGI) (Grigoriev et al. 2012). Outlier samples with <1,000,000 reads, more than 100,000,000 reads, or <70% of their total reads were removed from further analysis. Genes with <15 in 75% of samples were removed based on WGCNA guidelines (Langfelder and Horvath 2008), followed by variance stabilization normalization using the DESeq2 package (Love et al. 2014). The GEM for Arabidopsis was previously created using the same methodology and is publicly available: https://zenodo.org/records/10183151.

### Sample annotations

Sample information was retrieved from NCBI Biosample (Barrett et al. 2012) using the BioSampleParser tool (Limeta 2020). This annotation data was then manually evaluated to classify each Brachypodium sample as either “seed,” “leaf,” “root,” “flower,” or “aboveground_tissue.” This same manual annotation was previously done with the Arabidopsis dataset (Hadish et al. 2023) with the exception that “aboveground_tissue” was not a category and “seedling” was a category. PCA plots using only the gene expression matrix were used to confirm the validity of the manual sample annotation grouping methodology.

### Correlation analysis and plot construction

Pearson's correlation analysis was performed on *AHL* genes using the cor() function from the R v4.1.2 (R Core Team 2021) package “stats.” Results of this analysis were plotted using the pheatmap package (Kolde 2018) in R with clustering of correlation using the hclust. The pheatmap package was also used to plot expression levels, with scaling by rows performed. A Pearson's correlation score of 1 indicates a perfect positive correlation, where 2 genes are expressed in a perfectly coordinated manner. A score of −1 represents a perfect negative correlation, meaning the genes have opposite expression patterns. A score of 0 signifies no linear relationship, suggesting the genes' expression is independent.

## Results

### Identification and characterization of AHLs in Brachypodium

A bioinformatic approach was employed to identify putative AHL proteins in Brachypodium. This involved utilizing protein sequences of 29 Arabidopsis AHLs as queries in BLASTp and HMMER searches against the Brachypodium genome database. This comparative analysis identified a total of 22 AHL members in Brachypodium, designated *Bd*AHL1 to *Bd*AHL29 based on their sequence homology to the Arabidopsis AHL protein family. Further characterization of these *Bd*AHLs revealed a range in gene length (852–4767 bp), protein length (262–451 aa), and predicted average molecular mass (25.98–46.78 kDa) (Table 1). Subcellular localization prediction analysis suggested nuclear localization for all *Bd*AHLs (Table 1).

**Table 1.** Gene identification and analyses of the AHL gene family in Brachypodium.

Gene name	Gene ID	Gene length (bp)	Protein length (aa)	Average mass (kDa)	Isoelectric point	Net charge (pH 7.4)	Predicted subcellular localization (Y=Yes, N=No)		
							Chloroplast	Mitochondria	Nucleus
BdAHL1	Bradi3g13871	3824	395	39.75	9.130	+5.307	N	N	Y
BdAHL2	Bradi3g02510	4767	375	37.35	8.986	+4.358	N	N	Y
BdAHL5	Bradi5g26720	3057	406	41.78	9.656	+11.702	N	N	Y
BdAHL8	Bradi3g34030	4184	434	44.50	9.490	+9.888	N	N	Y
BdAHL9	Bradi3g55950	4645	451	46.78	6.573	−4.489	N	N	Y
BdAHL10	Bradi3g40180	3927	369	36.82	10.199	+15.235	N	N	Y
BdAHL11	Bradi1g29110	3299	341	35.23	6.279	−5.128	N	N	Y
BdAHL12	Bradi1g29120	4197	337	35.39	6.282	−5.011	N	N	Y
BdAHL13	Bradi5g19900	3948	374	37.76	8.897	+5.174	N	N	Y
BdAHL15	Bradi2g30423	1461	322	31.87	6.029	−4.527	N	N	Y
BdAHL15L	Bradi1g66880	1995	284	27.97	6.521	−2.368	N	N	Y
BdAHL17	Bradi4g32490	2473	372	38.27	7.307	−0.377	N	N	Y
BdAHL19	Bradi4g38870	1609	263	26.56	6.774	−1.489	N	N	Y
BdAHL20	Bradi3g12530	1922	286	28.75	6.130	−4.480	N	N	Y
BdAHL20L	Bradi3g55720	876	292	29.46	8.627	+2.422	N	N	Y
BdAHL21	Bradi3g16390	3477	292	28.79	6.116	−4.599	N	N	Y
BdAHL23	Bradi5g19920	1866	326	32.81	6.042	−6.712	N	N	Y
BdAHL24	Bradi3g11600	2050	313	31.68	6.243	−5.536	N	N	Y
BdAHL26	Bradi1g51520	1734	361	35.73	6.290	−5.377	N	N	Y
BdAHL27	Bradi1g35720	2792	262	25.98	6.655	−1.575	N	N	Y
BdAHL28	Bradi2g61076	852	278	28.66	6.521	−2.707	N	N	Y
BdAHL29	Bradi3g53220	3227	338	32.88	6.447	−2.658	N	N	Y

### Phylogenetic analysis of the Brachypodium *AHL* gene family

**Fig. 1.**
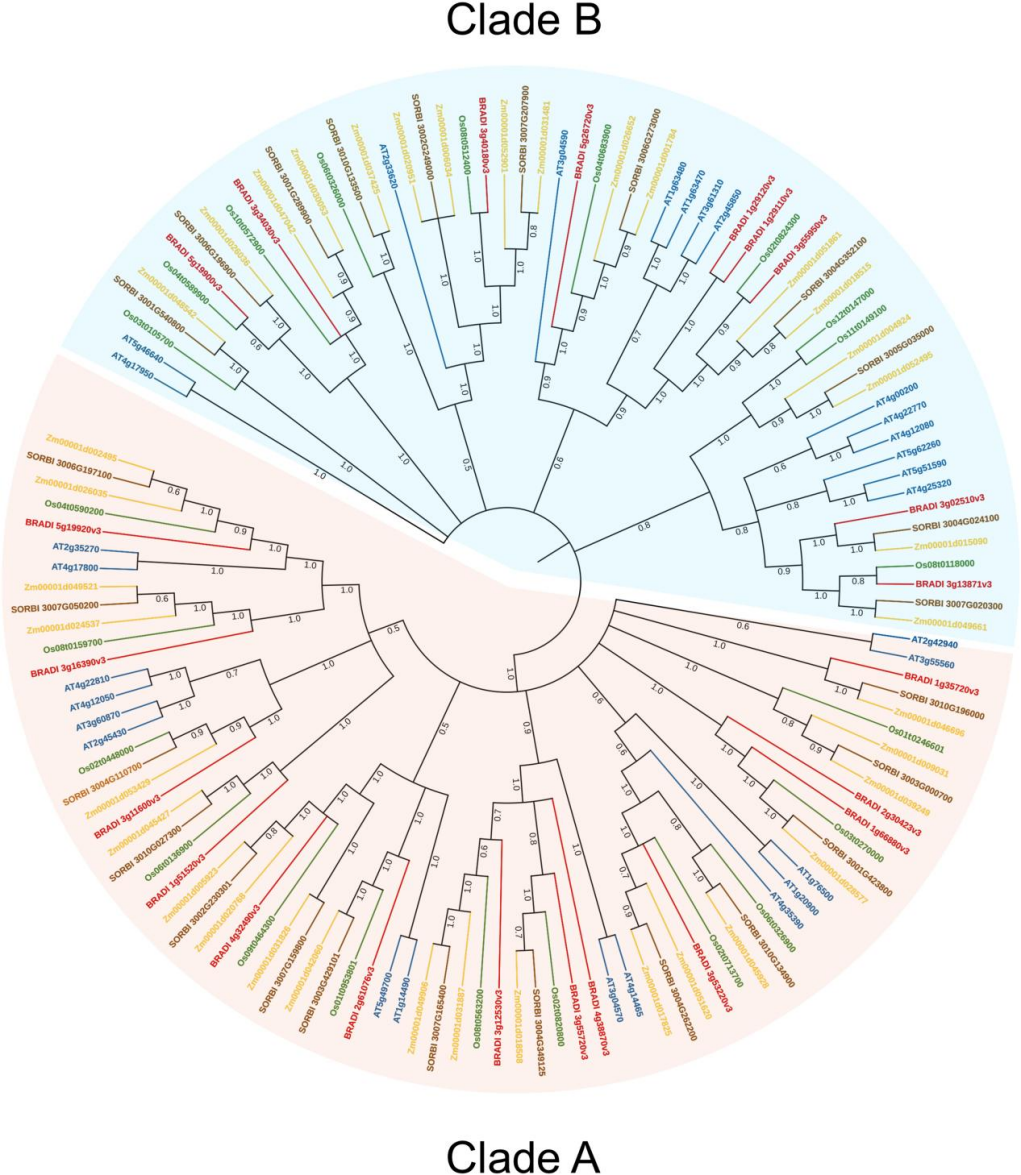
Phylogenetic relationships of the *AHL* gene family in grasses. A total of 135 protein sequences of the *AHL* gene family were identified from 5 species including *Arabidopsis thaliana* (blue), *Brachypodium distachyon* (red), *Zea mays* (yellow), *Sorghum bicolor* (brown), and *Oryza sativa* (green). All *AHL* genes were shaded in yellow (clade A) and blue (clade B) indicating the 2 different subgroups of *AHL* genes.

**Fig. 2.**
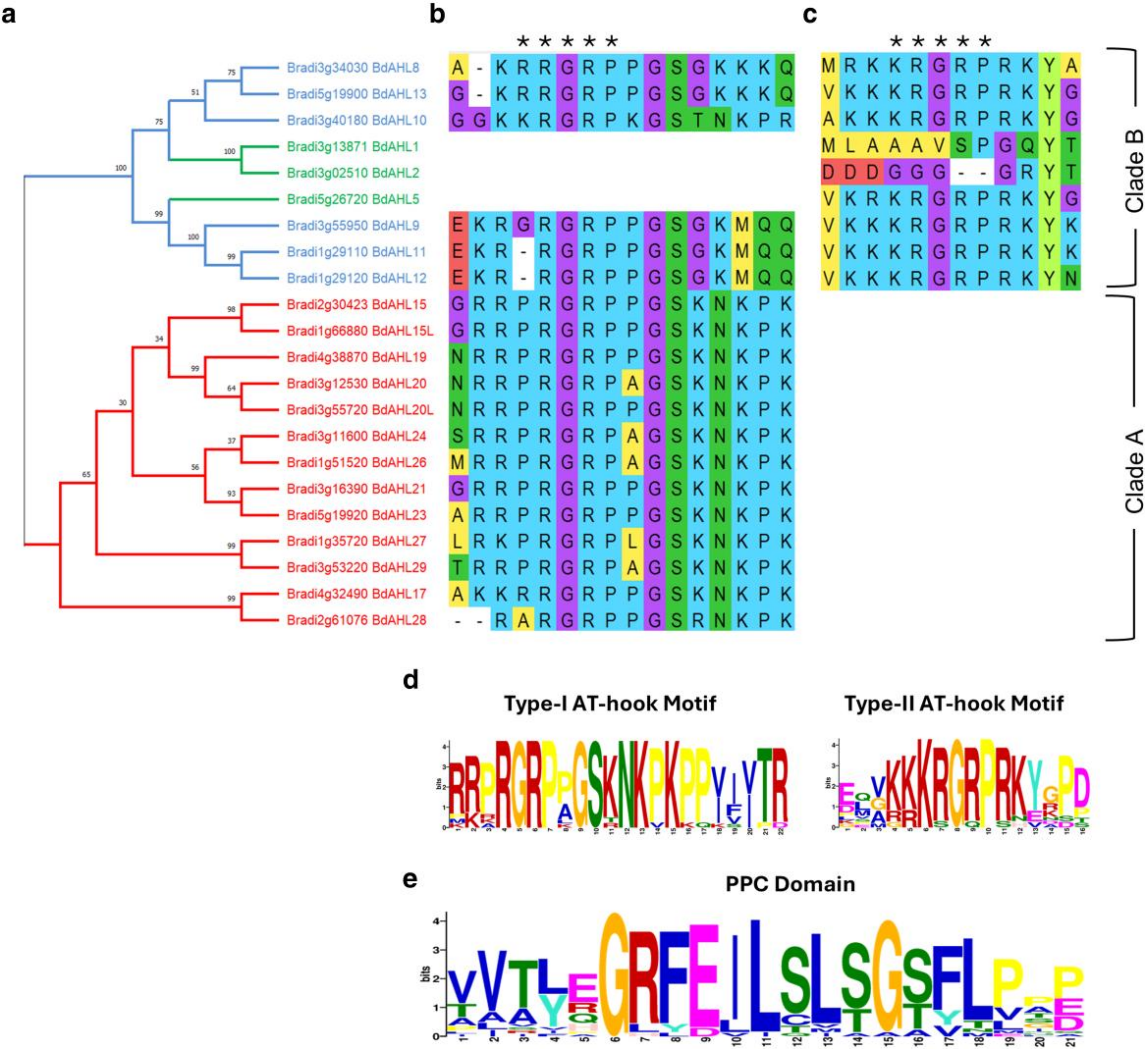
The *AHL* gene family in *B. distachyon*. a) Phylogenetic analysis of the *AHL* genes family using Maximum Likelihood and JTT matrix-based model. *AHL* genes containing only 1 Type-I AT-hook motif are shown in red. *AHL* genes containing 1 Type-II AT-hook motif are shown in green. *AHL* genes containing 2 AT-hook motifs (one of each type) are shown in blue. The amino acid sequence of the 2 AT-hook motifs identified: b) Type-I and c) Type-II. The stars indicate the highly conserved core regions of each AT-hook motif. Sequence logo visualization of d) Type-I and Type-II AT-hook motifs. e) Sequence logo analysis of both Type-A and -B PPC/DUF296 domains.

To elucidate the evolutionary relationships between Brachypodium AHL proteins and their counterparts in other plant species, a phylogenetic analysis was conducted. This involved constructing phylogenetic trees using multiple sequence alignments of AHL proteins from *B. distachyon*, *Oryza sativa* (rice), *Zea mays* (maize), *A. thaliana*, and *Sorghum bicolor* (sorghum). The analysis of a total of 135 genes revealed 2 distinct clades designated as clade A and clade B (Fig. 1). The distribution of AHL protein sequences within each clade showed a high degree of consistency, with homologs from the 5 species consistently forming distinct monophyletic groups that point to conserved evolutionary relationships. Clade A encompassed 15, 14, 20, 14, and 11 proteins from Arabidopsis, Brachypodium, maize, sorghum, and rice, respectively, while clade B contained 14, 9, 17, 11, and 10 proteins from the same species, respectively (Fig. 1). This observation suggests a strong evolutionary conservation of *AHL* genes across diverse plant species. When constructing a phylogeny encompassing all 22 Brachypodium AHL protein sequences (Fig. 2a), the evolutionary relationships between these genes were consistent with the Arabidopsis phylogeny published by Zhao et al. (2013). The homology observed between AHL proteins from different species strengthens the notion that family members within the same phylogenetic branch likely possess similar functions.

### Conserved motif prediction and gene structure analysis of AHL genes in Brachypodium

The AT-hook motifs displayed a highly conserved R–G–R core, classifying them as members of the AT-hook motif family, further categorized as type-I and type-II, respectively (Fig. 2b and 2c). Analysis using the MEME Suite 5.5.7 identified 3 conserved protein motifs within Brachypodium AHL proteins including both type-I and -II AT-hooks and PPC/DUF296 domain (Fig. 2d and 2e). Notably, the PPC/DUF296 domain was also identified containing the conserved G–R–F–E–I–L residues as previously reported in Arabidopsis (Zhao et al. 2014), but not in soybean (Bishop et al. 2020; Wang et al. 2021). Collectively, the phylogenetic analysis and motif prediction results indicated both the evolutionary overall consistency of *AHL* genes in both Arabidopsis and Brachypodium.

**Fig. 3.**
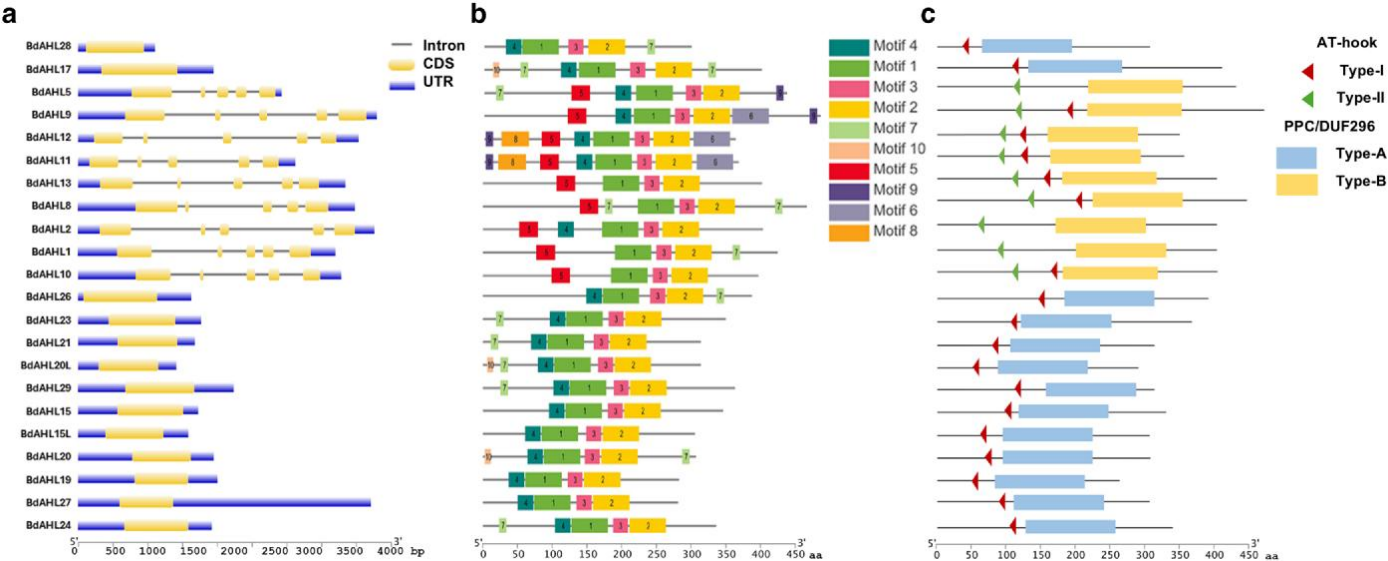
Gene structure, motifs, and functional domains of the Brachypodium *AHL* gene family. a) Gene structure analysis detailing introns, exons, and upstream/downstream UTR regions are shown as straight lines and yellow- and blue-bars, respectively. b) All identified motifs represented by color coded and numbered bars. c) Topology of 3 types of AHL proteins identified based on the combination of AT-hook motifs and PPC/DUF296 domains. The length and position of the conserved AT-hooks motifs (represented as red and green triangles, type-I and –II, respectively) and PPC/DUF296 domains (blue and yellow bars, type-A and -B, respectively) are shown.

Further investigation into Brachypodium gene structure demonstrated key features that are signatures of *AHL* gene architecture (Fig. 3). Brachypodium gene *BdAHL28* was identified as the shortest in length (852 bp) and BdAHL2 as the longest (4767 bp). Type-I genes were generally shorter than type-II and -III due to the lack of intronic regions. The number of introns and exons across *BdAHLs* also displayed significant diversity. Notably, a pattern emerged where type-I genes possessed only 1 exon and lacked introns, while type-II and type-III contained a greater number of both introns and exons (Fig. 3a). The motif analysis identified 10 conserved protein motifs within *Bd*AHL proteins including AT-hook type-I (motif 4) and type-II (motif 5). This analysis also identified 3 consecutive motifs (motif 1, 3, and 2) which reside in the PPC/DUF296 domain (Fig. 3b). These key functional motifs displayed conserved core regions classifying them as members of the AT-hook motif and PPC/DUF296 domain family (Fig. 3c). Different gene types detailing AT-hook and PPC/DUF296 domain types for each *Bd*AHL an evolutionary progression from type-I to types-II and -III, with a more complex genetic structure evolving from a simpler form. This finding aligns with previous reports on the *AHL* gene family in Arabidopsis (Zhao et al. 2014).

### Synteny and collinearity analysis of Brachypodium vs Arabidopsis *AHL* genes

**Fig. 4.**
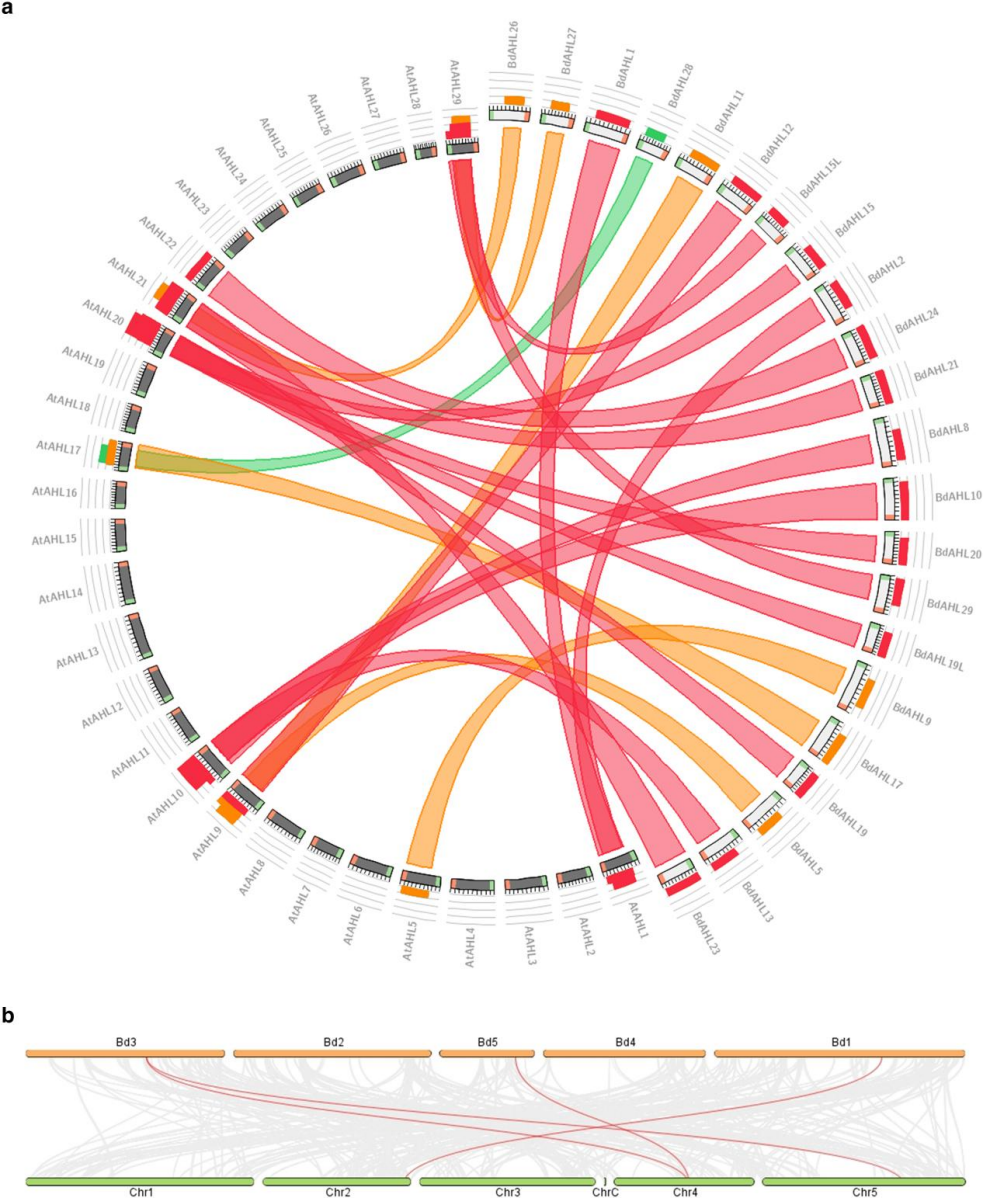
Homology, synteny, and collinearity analysis of *AHL* genes between Arabidopsis and Brachypodium. a) Sequence homology analysis using a Circoletto radial diagram linking the *Brachypodium distachyon* and *Arabidopsis thaliana AHL* orthologs with ribbons. The colors of the ribbons are relative to the best BLAST alignment score, with matches within 60% as green, within 80% as orange, and within 100% as red. White (*Bd*AHL) and black (*At*AHL) bands on the periphery of the diagram represent the protein sequences, with the start and end of the sequence shown as green and red blocks, respectively. Histogram on top of the diagrams count how many times each color has hit that specific part of the sequence. b) Synteny and dual collinearity plot between Brachypodium (chromosomes shown in orange) and Arabidopsis (chromosomes shown in green). Gray lines indicate syntenic blocks between both genomes, and the red lines indicate the syntenic gene pairs.

The sequence homology between Arabidopsis and Brachypodium *AHL* genes was visualized using a comparative diagram using the Circoletto tool. Ribbons in the diagram represent BLAST local alignment results, with Brachypodium sequences serving as queries. All *Bd*AHL proteins exhibited sequence identity exceeding 40% with Arabidopsis counterparts, with a majority demonstrating identity >81% (Fig. 4a). Syntenic genes, defined as those located on corresponding chromosomes across species, were further categorized as collinear when conserved gene order was also observed. Wang et al. (2012) identified 22,719 orthologous gene pairs between Arabidopsis and Brachypodium, of which 202 were determined to be collinear. Synteny and collinearity analysis of *AHL* genes revealed 4 collinear orthologous pairs (*BdAHL8* (Bd3)-*AtAHL8* (Chr5), *BdAHL8* (Bd3)*-AtAHL13* (Chr4), *BdAHL13* (Bd5)*-AtAHL13* (Chr4), *BdAHL26* (Bd1)*-AtAHL22* (Chr2)) between the 2 species (Fig. 4b), representing approximately 2% of the total collinear gene pairs identified by Wang et al. (2012).

### Protein interaction network analysis of Brachypodium and Arabidopsis AHLs

**Fig. 5.**
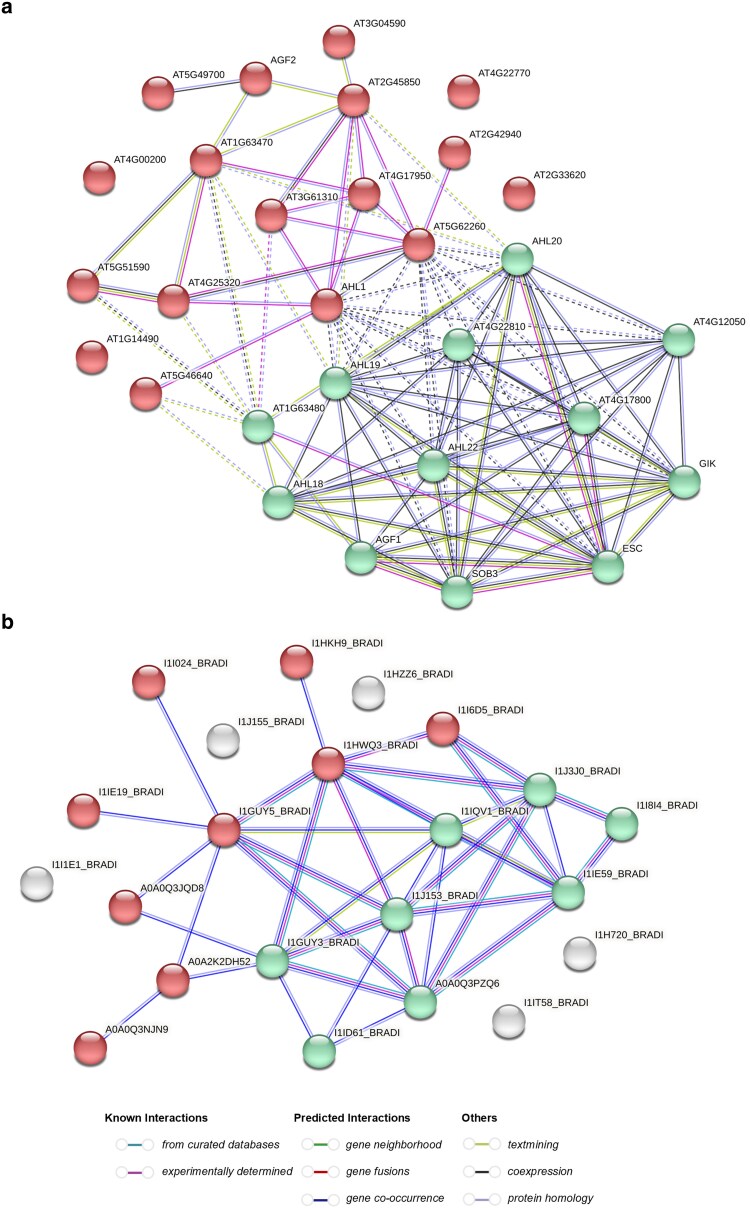
Arabidopsis and Brachypodium AHL protein–protein interaction network visualized by STRING 12.0. a) Protein–protein interaction network of all 29 *At*AHLs. b) Protein–protein interaction network of 22 *Bd*AHLs. The nodes indicate proteins, and edges indicate the interactions. Color saturation of the edges represents the confidence score of a functional association. Disconnected nodes are hidden, and only interactions with a confidence score of ≥0.4 are shown. A clustering function was employed to identify a defined number of clusters based on their centroids (input number of clusters was 2). Green nodes are clade A AHLs and red nodes are clade B AHLs. White nodes were proteins that were unassigned to cluster. Solid lines represent protein–protein associations whereas dotted lines represent network associations between clusters.

To examine AHL protein interactions, 2 independent protein–protein interaction networks were constructed including all identified AHLs: 22 *Bd*AHLs and 29 *At*AHLs (*P*-value: <1.0 × 10^−16^) (Fig. 5). Since pervious research has shown AHLs to interact with other AHLs within clades, there are few examples of experimentally confirmed cross-clade interactions (Dreze et al, 2011; Zhao et al. 2013). Protein interaction networks were constructed to explore all the known and predicted interactions within the AHL family in both Brachypodium and Arabidopsis. The *Bd*AHL network resulted in 22 nodes and 38 edges with an average node degree of 3.45 and average local clustering coefficient of 0.499. The *At*AHL network resulted in 29 nodes and 105 edges with an average node degree of 7.24 and average local clustering coefficient of 0.595. A k-means clustering method of 2 clusters was implemented on both interaction networks (Fig. 5). This clustering algorithm separated the nodes into 2 distinct modules. which resulted in the clustering of clade A and clade B AHLs in both networks. Not all nodes were assigned to a module in the Brachypodium network. The absence of a connection for a given node in Fig. 5b implies a lack of experimentally validated direct interactions with other Brachypodium AHLs within the STRING database. This suggests the protein may have different interaction partners, tissue-specific functions, or distinct regulatory roles compared to its Arabidopsis homolog.

### Gene duplication dating analysis

An analysis of synonymous (Ks) and nonsynonymous (Ka) substitution rates was conducted to investigate the evolutionary forces shaping 8 duplicated AHL genes. The Ka/Ks ratio provides a robust indicator of selective pressure, with values <1 signifying purifying (negative) selection, values >1 indicating positive selection, and values around 1 suggesting neutral selection. This analysis identified 8 paralogous *AHL* pairs in *Brachypodum* (Table 2). The Ka/Ks ratios for all the duplicated gene pairs were <1 implying these genes were under purifying selection (Table 2). This suggests that the gene duplications likely retained their ancestral functions after the duplication event. The Ks values were further employed to estimate the divergence time for each duplicated gene pair. The estimated duplication times ranged from 13.25 (*BdAHL*11-*BdAHL*12) to 161.57 million years ago (*BdAHL*8-*BdAHL*13) (Table 2), providing insights into the evolutionary history of these AHL genes.

**Table 2.** Ka/Ks analysis and estimated AHL gene duplication event time in Brachypodium.

No.	Paralogous Pair	Ka	Ks	Ka/Ks	Time (MYA)
1	Bradi4g32490(BdAHL17)–Bradi2g61076(BdAHL28)	0.495588042	0.955490638	0.518673885	73.49927987
2	Bradi1g29110(BdAHL11)–Bradi1g29120(BdAHL12)	0.027186123	0.172246156	0.157832973	13.2497043
3	Bradi3g13871(BdAHL1)–Bradi3g02510(BdAHL2)	0.343171039	0.999031864	0.343503597	76.84860489
4	Bradi3g34030(BdAHL8)–Bradi5g19900(BdAHL13)	0.306975705	2.100393965	0.146151489	161.5687666
5	Bradi2g30423(BdAHL15)–Bradi1g66880(BdAHL15L)	0.273219149	0.760887885	0.359079379	58.52983729
6	Bradi4g38870(BdAHL19)–Bradi3g12530(BdAHL20)	0.167939902	0.836637454	0.200731991	64.35672726
7	Bradi3g16390(BdAHL21)–Bradi5g19920(BdAHL23)	0.241843824	0.722844799	0.334572268	55.60344611
8	Bradi1g35720(BdAHL27)–Bradi3g53220(BdAHL29)	0.40796332	0.774761255	0.526566498	59.59701959

### *AHL* gene expression profile and gene coexpression analysis of Brachypodium vs Arabidopsis

**Fig. 6.**
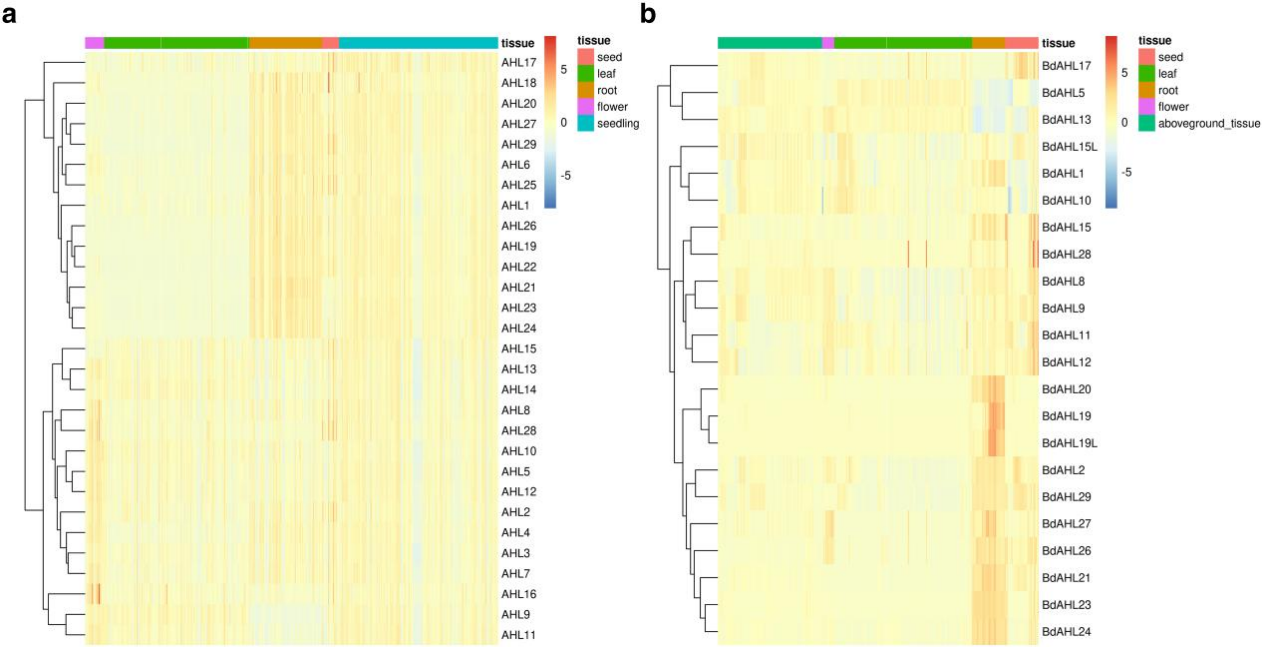
*AHL* gene expression levels across tissue type for a) Arabidopsis and b) Brachypodium. Each column represents a sample, and each row is an AHL gene. Samples were processed from publicly available NCBI datasets (Arabidopsis was downsampled to match Brachypodium). Relative expression level is shown from low expression (blue) to high expression (red) across all samples for each gene. Genes are organized based on expression level similarity. Only samples with annotation data are shown in this figure, with 1,153 Arabidopsis samples and 761 Brachypodium samples shown.

Gene expression for the Arabidopsis AHL family can be split into 2 categories, those which tend to be expressed in the root and seed/seedling stage (*At*AHL17, *At*AHL18, *At*AHL20, *At*AHL27, *At*AHL29, *At*AHL6, *At*AHL25, *At*AHL1, *At*AHL26, *At*AHL19, *At*AHL22, *At*AHL21, *At*AHL23, and *At*AHL24) and those which are more expressed in other tissues (often flower) (*At*AHL15, *At*AHL13, *At*AHL14, *At*AHL8, *At*AHL28, *At*AHL10, *At*AHL5, *At*AHL12, *At*AHL2, *At*AHL4, *At*AHL3, *At*AHL7, *At*AHL16, *At*AHL9, and *At*AHL11) (Fig. 6a). In general, clade A AHLs were upregulated in roots and downregulated in leaf and flower tissues in Arabidopsis. Gene expression for the Brachypodium data showed a similar pattern of some samples being upregulated in the roots and seed (*Bd*AHL20, *Bd*AHL19, *Bd*AHL19L, *Bd*AHL2, *Bd*AHL29, *Bd*AHL27, *Bd*AHL26, *Bd*AHL21, *Bd*AHL23, and *Bd*AHL24) whereas the remaining (*Bd*AHL17, *Bd*AHL5, *Bd*AHL13, *Bd*AHL15L, *Bd*AHL1, *Bd*AHL10, *Bd*AHL15, *Bd*AHL28, *Bd*AHL8, *Bd*AHL9, *Bd*AHL11, and *Bd*AHL12) were upregulated elsewhere. *Bd*AHL8, *Bd*AHL9, *Bd*AHL11, and *Bd*AHL12 appeared to be upregulated in flowers (Fig. 6b).

**Fig. 7.**
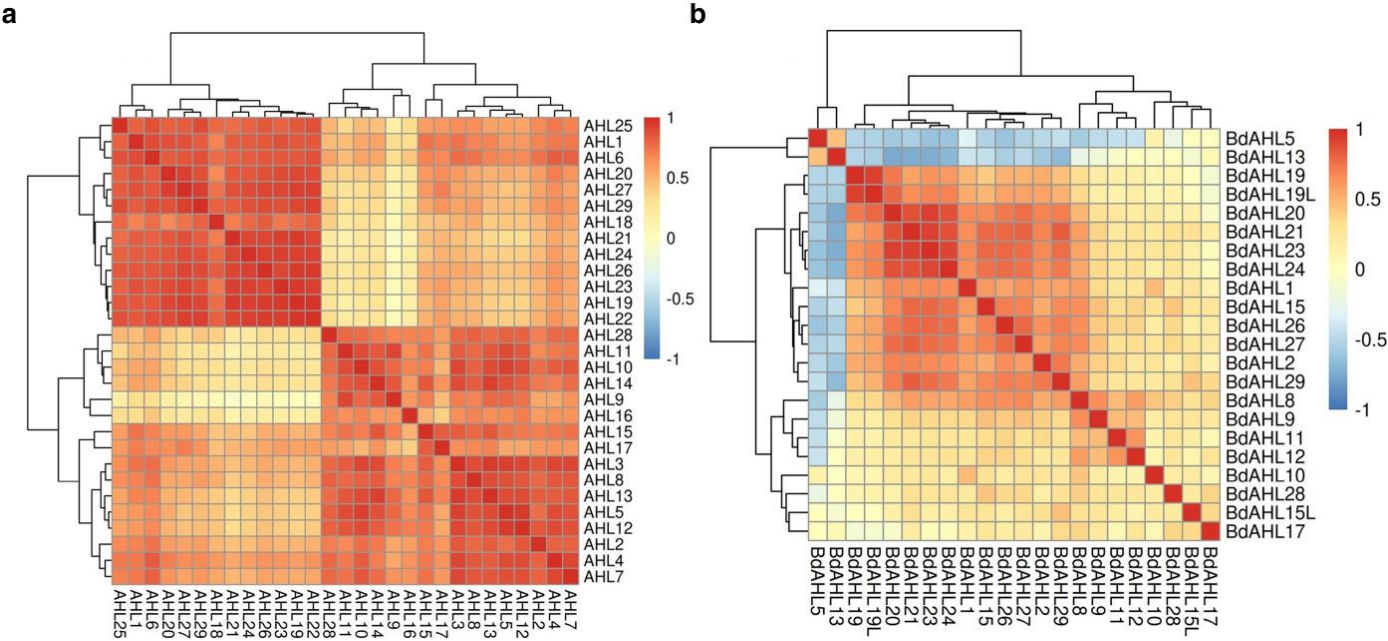
Coexpression correlation matrix for *AHL* genes in a) Arabidopsis and b) Brachypodium. Correlation is measured from −1 (perfect negative correlation) to 1 (perfect positive correlation). Colors represent the level of correlation from low to high.

A Pearson's correlation analysis was performed to investigate coexpression patterns among *AHL* genes in Arabidopsis and Brachypodium, with the results visualized in heatmaps. In Arabidopsis, a strong and distinct pattern of coexpression was observed within phylogenetic clades (Fig. 7a). Specifically, clade A *AHLs* exhibited a high positive correlation with other members of clade A, while clade B *AHLs* showed a similar highly positive correlation with other clade B members. In contrast, the coexpression patterns in Brachypodium were less pronounced (Fig. 7b). While most clade A *AHLs* showed a positive correlation with other members of the same clade, the magnitude of this correlation was not as strong or not correlated at all compared to those observed in Arabidopsis. A distinct pattern was noted for *Bd*AHL8, *Bd*AHL9, *Bd*AHL11, and *Bd*AHL12, which displayed slightly positive coexpression cluster. Furthermore, *Bd*AHL5 and *Bd*AHL13 exhibited a strong negative correlation with a majority of clade A *AHLs*.

## Discussion

The evolutionary history of the ancient *AHL* gene family throughout plant evolution displays a remarkable story of gene duplication followed by diversification and functional adaptation. The combined results of our analysis with prior research (Zhao et al. 2014) demonstrate that AHL proteins existed in early land plant lineages since their presence remains conserved in both mosses and flowering plants. The observed expansion of the number of AHL proteins in angiosperms compared to earlier plant lineages demonstrates their critical function in basic developmental processes throughout the plant kingdom. The noticeable growth of the *AHL* gene family within flowering plants demonstrates how gene duplication events can trigger evolutionary advancements, especially between eudicots and monocots. The field of comparative genomics enables researchers to reconstruct evolutionary events through the examination of orthologous and paralogous genes (for review see Levasseur and Pontarotti 2011). Evidence shows *AHL* genes underwent distinct expansion and functional divergence after the monocot–eudicot split which likely played a major role in creating unique developmental pathways within these plant clades. The existence of clade-specific *AHL* gene clusters indicates that gene duplication followed by subfunctionalization or neofunctionalization has been vital for adaptation to various ecological niches (Wendel 2000).

Understanding the evolutionary genetics of AHL proteins requires studying their functional domains which include the AT-hook motif and the Plant and PPC/DUF296 domain. The AT-hook motif enables minor groove DNA binding, which determines AHL proteins' affinity for AT-rich regions and controls their regulatory targets. Amino acid substitutions within this motif can modify DNA-binding affinity or specificity, which results in the recruitment of distinct downstream targets and causes functional divergence (Zhao et al. 2013). While the RGR motif is a hallmark of type-I and type-II AHLs, some family members like *Bd*AHL1 and *Bd*AHL2 may exhibit variations, truncations, or complete absence of this motif, likely due to evolutionary divergence or functional specialization. This variation in a conserved region may suggest a potential evolutionary divergence and/or distinct functional role. The PPC/DUF296 domain plays a vital role in protein–protein interactions through its capacity to construct both homo- and hetero-oligomers, which include trimers (Fujimoto et al. 2004; Seo and Lee 2021). Different mutations in this domain change interaction strength or specificity which affects functional complex formation and results in transcriptional regulation alterations. Domain-level variations play a critical role in enabling subfunctionalization or neofunctionalization after gene duplication events. Variations in the PPC domain can change how AHL proteins bind to specific cofactors, which results in altered downstream effects (Zhao et al. 2013). AHL complexes demonstrate the ability to attach to the minor groove of DNA and instigate conformational shifts which bring distal regulatory elements together to underline their transcriptional regulatory functions (Huth et al. 1997; Zhao et al. 2013). Understanding the evolutionary dynamics of these domains through amino acid substitution rates and selection pressures analysis proves essential to uncover the processes leading to *AHL* gene family diversification.

Brachypodium serves as a valuable model plant for the Pooideae grass subfamily. The importance of AHL proteins in plant growth, development, and stress tolerance has been established in Arabidopsis, maize, rice, cotton, and soybean. However, no Brachypodium AHL proteins have been identified to date. This study conducted a genome-wide analysis of Brachypodium AHLs, revealing 22 *Bd*AHLs. Phylogenetic analysis grouped *Bd*AHLs into 2 clades (A and B) and 3 types (I, II, and III), mirroring findings in other land plants (Supplementary Table 2). PPC/DUF296 domains potentially interact with each other or with other transcription factors, regulating transcriptional activation, suggesting diverse functions for *Bd*AHLs. Notably, a PPC/DUF296 domain was identified in *Bd*AHL proteins, which was absent in soybean AHLs. The notable absence of the conserved PPC/DUF296 domain in some soybean AHLs points to a significant evolutionary divergence in this gene family. This structural variation may imply alternative protein interaction mechanisms or distinct functional specializations that have evolved to fulfill unique roles in the soybean lineage, differing from their counterparts in monocots and Arabidopsis. Consequently, this observation provides a compelling hypothesis for the functional diversification of AHL proteins across other plant species.

*Bd*AHLs were predicted to localize exclusively in the nucleus. AHL proteins mainly accumulate in the nucleus, but they also exist in various other cellular compartments (Bishop et al. 2020; Wang et al. 2021; Chen et al. 2024). The gene family displays various functional possibilities. The transcriptional regulation function of AHL proteins explains their common presence within the nucleus. Research evidence demonstrates that these proteins exist in multiple cellular compartments besides the nucleus. The presence of AHL proteins in various cellular compartments shows they serve multiple functions beyond transcriptional regulation.

Uneven distribution of *Bd*AHL gene families across chromosomes and collinearity analysis indicated multiple gene duplication events within the Brachypodium genome, suggesting functional gain and loss through this process. The collinear relationships of *AHL* genes between Brachypodium and Arabidopsis suggest these genes were present in a common ancestor and have been functionally conserved since their divergence. This genomic synteny indicates that gene duplication events occurred before the 2 species diverged, providing a foundation for comparative genomics to infer the roles of uncharacterized genes.

Previous research has linked *AHL* genes to various stress responses. Cis-elements in promoters are known to influence plant growth, development, and stress responses. Anaerobic environments hinder root development and damage epidermal cells, increasing susceptibility to pathogens. Studies in grape and soybean have shown that *AHL* gene promoters contain light, hormone, and stress response elements, suggesting a role for *AHL* genes in these processes across Brachypodium and other plant species. The *AHL* gene family is prevalent in plants and plays a vital role in regulating flower, hypocotyl, root, and leaf development. To understand *Bd*AHL expression patterns, the relative expression levels of 22 *Bd*AHLs were compared to those of 29 *At*AHLs across different tissues. *Bd*AHLs displayed higher expression in flowers and roots compared to other tissues. Specific *AHL* genes, like *AHL3*, *AHL4*, *AHL18*, and *OsAHL1*, have been linked to root development, while *DP1*, *AHL16/TEK*, *AHL20*, *AHL21/GIK*, *AHL22*, *AHL23*, *AHL27*, and *BAF1* are associated with flower organ development. This suggests a crucial role for *Bd*AHLs in Brachypodium flower and root development, like the functions of AHLs in other plant species, such as *Dc*AHLc1, which is essential for storage root development in carrot. Our research identified and characterized *Bd*AHLs, providing insights into their potential functions in Brachypodium development and stress responses. Further research is needed to elucidate the specific roles of *Bd*AHLs in these processes.

Subsequent studies must investigate how distinct AHL paralogs function across various plant families. The combination of phylogenetic analyses with gene expression profiling and functional genomics approaches can help reveal the complete picture of how evolutionary forces shaped the *AHL* gene family. The examination of regulatory networks that include AHL proteins can reveal their impact on plant development and adaptation. Research on how AHL functions evolved through convergent evolution in response to similar environmental pressures across different plant lineages reveals important aspects of the evolutionary adaptability of this ancient gene family.

In this study, a total of 22 *AHL* genes were identified in Brachypodium, and they unevenly distributed on 5 chromosomes. The phylogenetic tree divided these genes into 2 clades and 3 types based on the AT-hook motif and PPC/DUF296 domain. The results from this study will contribute to the advancement of monocot crop genomics research which can frequently be hindered by large genome sizes and polyploidy. While rice is an attractive system for grass genomics due to its small genome size and available genome sequence, it is not particularly well-suited as a robust model system for all grass crops. There is growing interest to elucidate the mechanisms by which *AHL* genes affect overall growth and development not only in Arabidopsis but also in a monocot system. Identifying novel protein interactions, as well as characterizing their gain- and loss-of-function phenotypes, has the potential to improve our understanding of the significant roles that AHLs play in both Arabidopsis and Brachypodium. Phenotypic analysis in Arabidopsis thus far has exhibited favorable results when seen through the scope of crop biotechnology. The improvement of economically important plant species can be visualized when examining the roles AHLs have in Arabidopsis. Our goal was to translate Arabidopsis research, involving AHLs and their effect on seedling development and flowering time, into Brachypodium may contribute to our general understanding of AHLs across angiosperms. Thus, exploring AHL gene function in monocots is a significant step forward for research on this ancient gene family.

## Supplementary Material

jkaf275_Supplementary_Data

## Data Availability

No new sequencing was performed for this project. The list of reanalyzed SRA numbers, the normalized datasets and intermediary files used in this study are publicly available on Zenodo at https://doi.org/10.5281/zenodo.13328785. These intermediary files were originally produced for the study Hadish et al. 2023. All other data and analysis necessary for the replication of this current study's conclusions are presented within the text, figures, and tables. Supplemental material available at G3 online.

## References

[jkaf275-B1] Bailey TL, Johnson J, Grant CE, Noble WS. 2015. The MEME suite. Nucleic Acids Res. 43:W39–W49. 10.1093/nar/gkv416. 25953851 PMC4489269

[jkaf275-B2] Barrett T et al 2012. BioProject and BioSample databases at NCBI: facilitating capture and organization of metadata. Nucleic Acids Res. 40:D57–D63. 10.1093/nar/gkr1163. 22139929 PMC3245069

[jkaf275-B3] Berardini TZ et al 2015. The Arabidopsis information resource: making and mining the “gold standard” annotated reference plant genome. Genesis. 53:474–485. 10.1002/dvg.22877. 26201819 PMC4545719

[jkaf275-B4] Bevan MW, Garvin DF, Vogel JP. 2010. *Brachypodium distachyon* genomics for sustainable food and fuel production. Curr Opin Biotechnol. 21:211–217. 10.1016/j.copbio.2010.03.006. 20362425

[jkaf275-B5] Bishop EH, Kumar R, Luo F, Saski C, Sekhon RS. 2020. Genome-wide identification, expression profiling, and network analysis of AT-hook gene family in maize. Genomics. 112:1233–1244. 10.1016/j.ygeno.2019.07.009. 31323298

[jkaf275-B6] Blanc G, Wolfe KH. 2004. Widespread paleopolyploidy in model plant species inferred from age distributions of duplicate genes. Plant Cell. 16:1667–1678. 10.1105/tpc.021345. 15208399 PMC514152

[jkaf275-B7] Braidwood L, Breuer C, Sugimoto K. 2014. My body is a cage: mechanisms and modulation of plant cell growth. New Phytol. 201:388–402. 10.1111/nph.12473. 24033322

[jkaf275-B8] Bray NL, Pimentel H, Melsted P, Pachter L. 2016. Near-optimal probabilistic RNA-seq quantification. Nat Biotechnol. 34:525–527. 10.1038/nbt.3519. 27043002

[jkaf275-B9] Chen W, Chen L, Cui L, Liu Z, Yuan W. 2024. Genome-wide analysis of radish AHL gene family and functional verification of RsAHL14 in tomato. Front Plant Sci. 15:1401414. 10.3389/fpls.2024.1401414. 38872889 PMC11169806

[jkaf275-B10] Chen C et al 2020. TBtools: an integrative toolkit developed for interactive analyses of big biological data. Molecular Plant. 13:1194–1202. 10.1016/j.molp.2020.06.009 32585190

[jkaf275-B11] Cui L et al 2006. Widespread genome duplications throughout the history of flowering plants. Genome Res. 16:738–749. 10.1101/gr.4825606. 16702410 PMC1479859

[jkaf275-B12] Darzentas N . 2010. Circoletto: visualizing sequence similarity with circos. Bioinformatics. 26:2620–2621. 10.1093/bioinformatics/btq484. 20736339

[jkaf275-B13] Draper J et al 2001. *Brachypodium distachyon*. A new model system for functional genomics in grasses. Plant Physiol. 127:1539–1555. 10.1104/pp.010196. 11743099 PMC133562

[jkaf275-B14] Dreze M et al 2011. Evidence for network evolution in an *Arabidopsis* interactome map. Science. 333:601–607. 10.1126/science.1203877. 21798944 PMC3170756

[jkaf275-B15] Fujimoto S et al 2004. Identification of a novel plant MAR DNA binding protein localized on chromosomal surfaces. Plant Mol Biol. 56:225–239. 10.1007/s11103-004-3249-5. 15604740

[jkaf275-B16] Goodstein DM et al 2012. Phytozome: a comparative platform for green plant genomics. Nucleic Acids Res. 40:D1178–D1186. 10.1093/nar/gkr944. 22110026 PMC3245001

[jkaf275-B17] Grigoriev IV et al 2012. The genome portal of the department of energy joint genome institute. Nucleic Acids Res. 40:D26–D32. 10.1093/nar/gkr947. 22110030 PMC3245080

[jkaf275-B18] Guo C et al 2023. Phylogenomics and the flowering plant tree of life. J Integr Plant Biol. 65:299–323. 10.1111/jipb.13415. 36416284

[jkaf275-B19] Hadish JA et al 2022. GEMmaker: process massive RNA-seq datasets on heterogeneous computational infrastructure. BMC Bioinformatics. 23:1–11. 10.1186/s12859-022-04629-7. 35501696 PMC9063052

[jkaf275-B20] Hadish JA, Honaas LA, Ficklin SP. 2023. Predicting phenotypic traits using a massive RNA-seq dataset [preprint]. bioRxiv. 10.1101/2023.12.05.570195.

[jkaf275-B21] Huth JR et al 1997. The solution structure of an HMG-I(Y)-DNA complex deﬁnes a new architectural minor groove binding motif. Nat Struct Biol. 4:657–665. 10.1038/nsb0897-657. 9253416

[jkaf275-B22] International Brachypodium Initiative . 2010. Genome sequencing and analysis of the model grass *Brachypodium distachyon*. Nature. 463:763–768. 10.1038/nature08747. 20148030

[jkaf275-B23] Jones P et al 2014. InterProScan 5: genome-scale protein function classification. Bioinformatics. 30:1236–1240. 10.1093/bioinformatics/btu031. 24451626 PMC3998142

[jkaf275-B24] Josuran R, Grolimund J, Thurnheer A, Iten M. 2025. Prot pi: Protein analysis tool. Version 2.2.29.152. Zurich University of Applied Sciences (ZHAW). https://www.protpi.ch/Calculator/ProteinTool.

[jkaf275-B25] Kolde R. 2018. Package “pheatmap” (Version 1.0.12). [cited 2025 Jun 16]. https://cran.r-project.org/web/packages/pheatmap/pheatmap.pdf.

[jkaf275-B26] Langfelder P, Horvath S. 2008. WGCNA: an R package for weighted correlation network analysis. BMC Bioinformatics. 9:559. 10.1186/1471-2105-9-559. 19114008 PMC2631488

[jkaf275-B27] Lee TH, Tang H, Wang X, Paterson AH. 2012. PGDD: a database of gene and genome duplication in plants. Nucleic Acids Res. 41:D1152–D1158. 10.1093/nar/gks1104. 23180799 PMC3531184

[jkaf275-B28] Levasseur A, Pontarotti P. 2011. The role of duplications in the evolution of genomes highlights the need for evolutionary-based approaches in comparative genomics. Biol Direct. 6:11. 10.1186/1745-6150-6-11. 21333002 PMC3052240

[jkaf275-B29] Limeta A. 2020. BioSampleParser. [cited 2025 Jun 16]. https://github.com/angelolimeta/BioSampleParser.

[jkaf275-B30] Liu S et al 2014. The Brassica oleracea genome reveals the asymmetrical evolution of polyploid genomes. Nat Commun. 5. 10.1038/ncomms4930. PMC4279128 24852848

[jkaf275-B31] Love MI, Huber W, Anders S. 2014. Moderated estimation of fold change and dispersion for RNA-Seq data with DESeq2. Genome Biol. 15:550. 10.1186/s13059-014-0550-8. 25516281 PMC4302049

[jkaf275-B32] Martinčová M, Soukup A. 2025. Twenty years of AT-HOOK MOTIF NUCLEAR LOCALIZED (AHL) gene family research–their potential in crop improvement. Curr Plant Biol. 42:100460. 10.1016/j.cpb.2025.100460.

[jkaf275-B33] Matsushita A, Furumoto T, Ishida S, Takahashi Y. 2007. AGF1, an AT-hook protein, is necessary for the negative feedback of *AtGA3ox1* encoding GA 3-oxidase. Plant Physiol. 143:1152–1162. 10.1104/pp.106.093542. 17277098 PMC1820926

[jkaf275-B34] NCBI Resource Coordinators . 2016. Database resources of the national center for biotechnology information. Nucleic Acids Res. 44:D7–D19. 10.1093/nar/gkv1290. 26615191 PMC4702911

[jkaf275-B35] Ng K-H, Yu H, Ito T, Weigel D. 2009. AGAMOUS controls GIANT KILLER, a multifunctional chromatin modifier in reproductive organ patterning and differentiation. PLoS Biol. 7:e1000251. 10.1371/journal.pbio.1000251. 19956801 PMC2774341

[jkaf275-B36] R Core Team . 2021. R: a language and environment for statistical computing. R Foundation for Statistical Computing.

[jkaf275-B37] Seo M, Lee J-Y. 2021. Dissection of functional modules of AT-HOOK MOTIF NUCLEAR LOCALIZED PROTEIN 4 in the development of the root xylem. Front Plant Sci. 12. 10.3389/fpls.2021.632078. PMC8056045 33889164

[jkaf275-B38] Song W et al 2014. Delineation of plant caleosin residues critical for functional divergence, positive selection and coevolution. BMC Evol Biol. 14:1–14. 10.1186/1471-2148-14-124. PMC4057654 24913827

[jkaf275-B39] Sperschneider J et al 2017. LOCALIZER: subcellular localization prediction of both plant and effector proteins in the plant cell. Sci Rep. 7:44598. 10.1038/srep44598. 28300209 PMC5353544

[jkaf275-B40] Szklarczyk D et al 2023. The STRING database in 2023: protein–protein association networks and functional enrichment analyses for any sequenced genome of interest. Nucleic Acids Res. 51:D638–D646. 10.1093/nar/gkac1000. 36370105 PMC9825434

[jkaf275-B41] Tamura K, Stecher G, Kumar S. 2021. MEGA 11: molecular evolutionary genetics analysis version 11. Mol Biol Evol. 38:3022–3027. 10.1093/molbev/msab120. 33892491 PMC8233496

[jkaf275-B42] Thomas PD et al 2003. PANTHER: a library of protein families and subfamilies indexed by function. Genome Res. 13:2129–2141. 10.1101/gr.772403. 12952881 PMC403709

[jkaf275-B43] Wang M, et al 2021. Genome-wide identification and expression analysis of the AT-hook motif nuclear localized gene family in soybean. BMC Genomics. 22:361. 10.1186/s12864-021-07687-y. 34006214 PMC8132359

[jkaf275-B44] Wang Y et al 2012. MCScanX: a toolkit for detection and evolutionary analysis of gene synteny and collinearity. Nucleic Acids Res. 40:e49–e49. 10.1093/nar/gkr1293. 22217600 PMC3326336

[jkaf275-B45] Wendel JF . 2000. Genome evolution in polyploids. In: Henry RJ, editor. Plant molecular evolution. CABI Publishing. p. 225–249. 10688139

[jkaf275-B46] Yun J, Kim YS, Jung JH, Seo PJ, Park CM. 2012. The AT-hook motif-containing protein AHL22 regulates flowering initiation by modifying FLOWERING LOCUS T chromatin in Arabidopsis. J Biol Chem. 287:15307–15316. 10.1074/jbc.M111.318477. 22442143 PMC3346147

[jkaf275-B47] Zhao J, Favero DS, Peng H, Neff MM. 2013. *Arabidopsis thaliana* AHL family modulates hypocotyl growth redundantly by interacting with each other via the PPC/DUF296 domain. Proc Natl Acad Sci U S A. 110:E4688–E4697. 10.1073/pnas.1219277110. 24218605 PMC3845178

[jkaf275-B48] Zhao J, Favero DS, Qiu J, Roalson EH, Neff MM. 2014. Insights into the evolution and diversification of the AT-Hook motif nuclear localized gene family in land plants. BMC Plant Biol. 14:1–19. 10.1186/s12870-014-0266-7. PMC4209074 25311531

